# The comparison of outcomes of surgically treated bilateral temporomandibular joint disorder in different groups: A retrospective study

**DOI:** 10.4317/medoral.18164

**Published:** 2012-08-28

**Authors:** Birkan T. Ozkan, Hannu Pernu, Kyosti Oikarinen, Aune Raustia

**Affiliations:** 1 DDS, PhD. Assistant Professor, University of Yuzuncuyil, Faculty of Dentistry, Department of Oral&Maxillofacial Surgery, Van, TURKIYE; 2 DMD. Assistant Professor, University of Oulu, Instıtute of Dentistry, Department of Oral&Maxillofacial Surgery, Oulu University Hospital, Oulu, FINLAND; 3 DMD. Professor, University of Oulu, Instıtute of Dentistry, Department of Oral&Maxillofacial Surgery, Oulu University Hospital, Oulu, FINLAND; 4 DDS, PhD. Professor, University of Oulu, Instıtute of Dentistry, Department of Prosthetic Dentistry and Stomatognathic Physiology, Oulu University Hospital, Oulu, FINLAND

## Abstract

Objectives: The main purpose of this study was to determine the prognosis and outcomes of the patients with bilateral temporomandibular disorder which underwent bilateral temporomandibular joint surgery in a consecutive number of patients in a retrospective study. Study Design: Sixty five patients with 130 bilateral TMJ were included the study with the selection from consecutive 256 TMJ patients who were treated with open surgery who do not respond to conservative treatment. 65 patients were divided in to 3 main groups according to the clinical diagnosis of bilateral TMJ site. In the first group comprised 29 patients with 48 TMJ, the clinical diagnosis was bilaterally presence of anterior disc displacement with reduction (ADDR). In the second group comprised 19 patients with 26 TMJ, bilateral presence of TMD consisted of anterior disc displacement without reduction (ADDNR) on both site. In the third group comprised 27 patients with 46 TMJ, bilaterally presence of TMD consist of ADDR on one site and ADDNR on another site. The patients in three different groups were operated either high condylectomy alone or high condylectomy with additional surgical procedures. Results: In the evaluation of pain relief, clicking, crepitation, headache, marked improvement was determined in all groups, but it was statistically insignificant in the comparison of 3 groups. Slight increase in maximal mouth opening was determined in the mean values of the 3 groups and also in the comparison of 3 groups it was not statistically significant. Conclusions: These similar succesfull outcomes of bilateral TMD with the respect of TMJ surgical procedures were obtained in 3 main groups although different diagnosis on the patients’ groups waspresent.

** Key words:**Temporomandibular joint, prognosis, retrospective studies.

## Introduction

Temporomandibular disorder (TMD) is a term which summarizes anterior disc displacement with reduction (ADDR) or anterior disc displacement without reduction (ADDNR), perforation of the articular disc or of the retrodiscal tissue and various degene-tartive changes of the disc and/or the articulating surfaces (1). The subjective presentation of patients with TMD can range from limited function secondary to increased pain levels, such as the reduced ability to chew hard food, to limited mouth-opening, and limitations of the excursive movements of the joint (2). More than 50 % of the population exhibit symptoms of TMD and up to 30 % of these people are in need of stomatognathic treatment. Most TMD patients can be succesfully treated by conservative treatment modalities such as counseling, splint therapy, mandbibular exercies, and occlusal adjustement or a combination of these, but some patients (10-20 %) symptoms persist in spite of conservative treatment (3). But neverthless, in some cases (5-10 %) who do not respond to conservative treatment may benefit from surgical treatment (4).

These are various surgical treatment modalities with different diagnosis for temporomandibular joint (TMJ) disorder. The following five main methods can be used a) disc repositioning b) condyler shaving (high condylectomy) c) condylectomy d) discectomy with or without implants; and e) arthroscopic surgery (5).

There is no consensus on the choice of the surgical procedure for the treatment of temporomandibular disorders so far. This general feeling is confirmed in a recent meta-analaysis in which no differences were found among the surgical procedures (6).

The hypothesis in this study whether the bilateral TMJ cases behave differently than unilateral cases and also whether any difference in prognosis present for bilateral cases with different TMJ diagnosis operated similiar surgical techniques.

The main purpose of this study was to determine the prognosis and outcomes of the patients with bilateral TMD which underwent bilateral TMJ surgery in a consecutive number of patients in a retrospective study and also to compare the groups containing as the first group bilateral ADDR, the second group with bilateral ADDNR, as the third group with ADDR on one side and ADDNR on another side.

## Patient and Methods

Sixty five patients with 130 TMJ were included the study with the selection from consecutive 256 TMJ (unilateral and bilaterally operated TMJ) patients who were treated with open surgery who do not respond to conservative treatment between 1987 and 2000 in the Oral and Maxillofacial Department, Oulu University Hospital. The study was approved by the Ethical Committee of the Northern Ostrobothnia Hospital District.

On the basis of selection criteria, patients with previously underwent TMJ surgery orthognathic surgery and/or other different joint artridities, arthroscopies, unilateral involvement of TMJ surgery and presence of known connective tissue/autoimmune diseases were excluded from the study. Bilateral joint involvement with internal derangement, TMJ’s with both reducing and non- reducing discs, intolerable pain after standard medical treatment and consecutive patients were included the study.

Before surgery all patients had received various conservative treatment included occlusal adjusment, physiotherapy, medication with analgesics and NSAI drugs, occlusal splints, patient education, acupuncture and prosthetic rehabilitation, local corticosteroid injection.

Age, gender, chief complaints, presence of history of trauma and/or systemic diseases, duration of symptoms, conservative treatment type before surgery, clinical diagnosis, MRI/ CT findings, operated TMJ side, operation technique, surgical findings during operation, follow-up period and outcome of treatment were obtained from patients files and recorded.

Definitive TMJ diagnosis before surgery was based on clinical, MRI and/or CT examination. The main indications for surgery were consistent and intolerable TMJ and facial pain, abnormal sounds on TMJ, locking of the TMJ and restricted mouth opening despite conservative treatment.

A total number of 65 patients with bilateral TMD were administered by the same surgeon (H.P) under general anesthesia and evaluated definitive diagnosis presurgical and postoperative follow-up examinations. Another researcher (B.T.O) collected all data of patient medical records and selected the patients who convenient for the inclusion criteria from the consecutive patients’ files. All were operated through high condylectomy with additional surgical procedure included: no additional surgery, releasing of adhesion, repositioning of disc, discectomy, osteoplasty.

Sixty five patients were divided in to 3 main groups according to the clinical diagnosis of bilateral TMJ site. In the first group comprised 29 patients with 48 TMJ, the clinical diagnosis was bilaterally presence of ADDR. Out of 29 patients, 22 patients underwent high condylectomy alone, 5 patients with high condylectomy plus discectomy, 2 patients with high condylectomy with reposition of disc. In the second group comprised 19 patients with 26 TMJ, bilateral presence of TMD consisted of ADDNR on both site. Out of 19 patients, 13 patients underwent high condylectomy alone, 2 patients with high condylectomy with reposition of disc, 2 patients with high condylectomy with discectomy, 2 patients with high condylectomy with osteoplasty. In the third group comprised 27 patients with 46 TMJ, bilaterally presence of TMD consist of ADDR on one site and ADDNR on another site. Out of 27 patients, 23 patients underwent high condylectomy alone, 4 patients with high condylectomy with discectomy.

High condylectomy was performed through a preauricular incision with a temporal extension which made 45º to the zygomatic arch, from the superior auriculocutaneous tissue, the superficial fascia, and the areolar fat tissue. Blunt dissection is carried out downwards, to a point 2cm above the malar arch where the deep temporalis fascia splits into two layers containing fat tissue. Subsequent to dissection completion, joint exposure was managed. After joint exposure a 2-3 mm section of the anterior-superior slope of the condyle was removed and the articular surface reshaped. The cortical edges were them smoothed by shaving. After high condylectomy procedure, if required, additional surgical procedures were administered.

Before and after TMJ operations, demographic data, maximal interincisal opening (vertical ROM) (measured with a ruler), presence or absence of pain on TMJ (yes/no), crepitation (on palpation with a finger or auscultation with a stetoscope), headache, clicking (on palpation with a finger or auscultation with a stetoscope), permanent of temporary facial paralysis, deviation, patient satisfaction, follow-up period variables were recorded from the patient files for each of the 3 study groups. All of the patients were clinically and radiographically assessed these initial and postoperative assessments through simply anectodal determinations. Patient satisfaction was determined by the surgeon through scoring (0= excellent, 1= good, 2= mild, 3= bad). Postoperatively, follow-up treatment included the use of analgesics for two to four weeks and mobilization exercises of the lower jaw, beginning the day after the operation. In order to eliminate the postsurgical open bite and probable changes in occlusion, occlusal adjustment and prosthetic rehabilitation, if required, were performed approximately six months after the operation. The mean values were calculated ( Table 1).

**Table 1:**
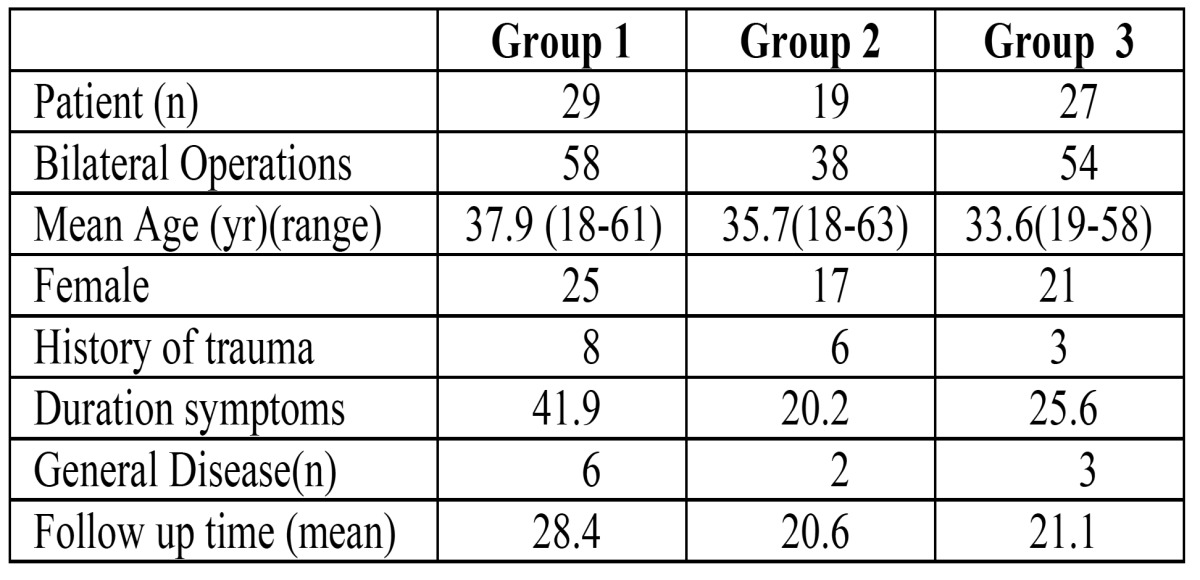
Demographic characteristics, duration of symptoms and history of trauma for 3 study groups.

The SAS system statistical software package was used to analyse the quantitive results obtained. Descriptive statistics was employed to calculate the mean, standard deviation, minimum and maximum values for different variables. Wilcoxon T test was used to compare the degree of mouth opening before and after surgical modalities in different patients groups.

In the comparison of preopaerative and postoperative symptoms, the McNemar’s and Binomial test were used. Duncan’s Multiple Range test was used to compare 3 groups. The level of statistical significance was taken as P≤ .05.

## Results

Descriptive statistics and demographic characteristics of the patients for 3 study groups were summarized in  Table 1 65 patients (130 TMJ) underwent bilateral TMJ surgery.

Specific documented evaluations for all these parameters evaluated and recorded at all presurgical and postsurgical evaluations.

The patients were followed up postoperatively for an average of 26.7 months (range 18 to 156 months). Duration of syptoms prior to conservative and surgical treatment was statistically not significant between groups. History of trauma of the first and second groups was statistically higher than the 3rd group. Female predilection was high percent in all groups.

**Figure 1:**
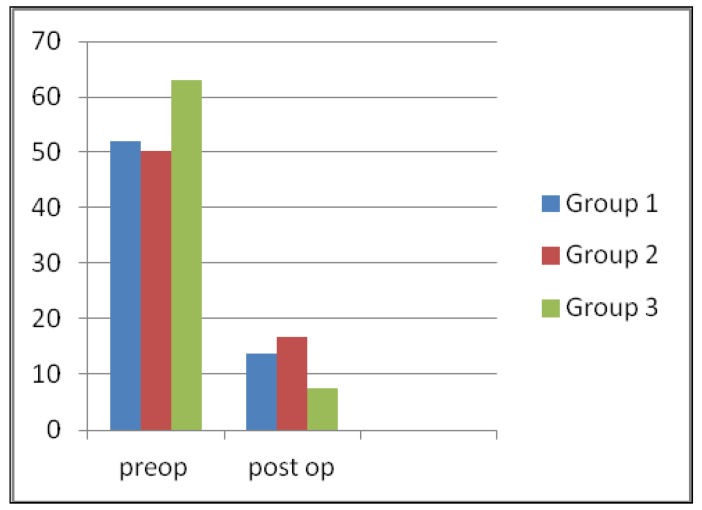
The percentage for presence of clicking preoperatively and postoperatively in 3 study groups.

**Figure 2:**
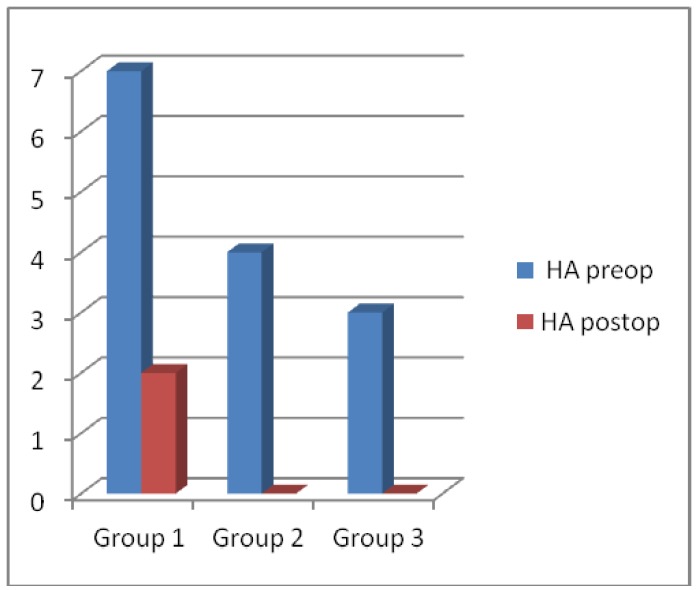
The percentage for presence of headache preoperatively and postoperatively in 3 study groups.

In the evaluation of presence of pain, clicking, crepitation, headache, marked improvement was determined and it was statistically significant in all groups, but it was statistically not significant in the comparison of 3 groups (Figs. 1,2).

**Figure 3:**
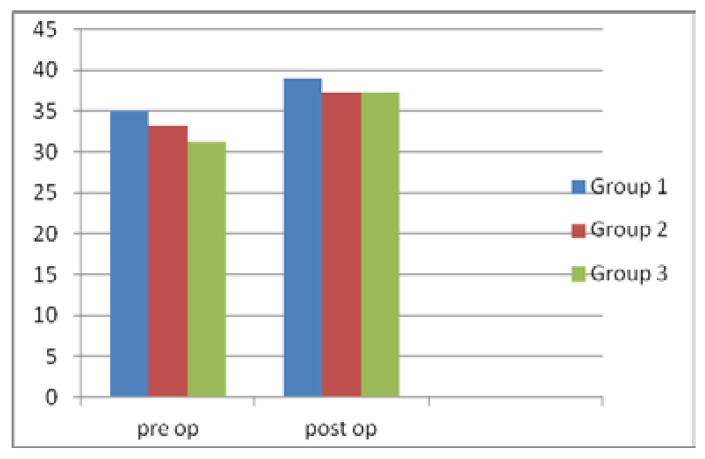
The amount of mean values for maximal mouth opening for 3 study groups preoperatively and postoperatively.

The improvement for maximal mouth opening postoperatively in the 1st, 2nd and 3rd group was 3.5mm, 4.1mm and 6.07mm, respectively. Slight increase in maximal mouth opening was determined in the mean values of the 3 groups and also in the com-parison of 3 groups it was not statistically significant (Fig. 3).

Deviation remained unchanged and no improvement was observed in the comparison of preoperatively and postoperatively in all groups.

Temporary facial paralysis was present in one patient in the second group and also facial paralysis in temporal region was present in 1 patient.

Patient satisfaction was high in all groups, but the difference between groups was statistically no significant.

All parameters improved with the exception of deviation in all groups’ patients.

## Discussion

Many disease processes are able to affect the TMJ, though four disorders either alone or in combination account for the immense majority of cases requiring medical attention: myofascial pain, ADDR, disc displacement without reduction ADDNR, and osteoarthrosis. Classically, one of the most important deficiencies in the study of TMD has been the lack of valid, reliable and reproducible criteria for the precise classification of the disorders (7). TMD pain is by far the most common reason patients seek treatment (8,9).

The American Academy of Orofacial Pain (AAOP) has published TMD diagnostic categories and criteria. The diagnostic categories are separated into TMJ masticatory muscle disorders and articular disorders which often accompanied by internal derangement, include noninflammatory and inflammatory arthropathies, growth disorders, and connective tissue disorders. Internal derangements are classically divided into two groups: ADDR and ADDNR. Treatment of patients with internal derangement of the TMJ typically begins with nonsurgical treatment modalities. Bite appliance therapy, diet modifications, nonsteroidal anti-inflammatory medications, muscle relaxants, moist heat or ice, and physical therapy have been found to be efficacious. Surgical intervention is typically employed only after failure of nonsurgical treatment objectives. Historically, clinicians have recognized that surgery for internal derangements should be reserved for patients with pain or dysfunction that is severe and disabling and is refractory to nonsurgical management. These conditions still form the basic indications for surgery (10).

Most of these cases demonstrate a reducing disk displacement, in which the disk represents a mobile mechanical obstacle and the condyle is not permanently restricted in its range of motion. Reduction refers to the ability of the condyle to negotiate around the disk. The disk’s recoil potential is minimal in the pathologic condition. The inferior surface of the disk is typically bulged and histologically is the site of increased proteoglycan deposition. If pain and dysfunction persist despite treatment of a coexistent parafunctional habit, surgery should be considered. These patients are best managed with open surgery and reduction of the obstructing portions of the articular disk. Diskoplasty, partial diskectomy, or full diskectomy may be performed, depending on the degree of disk atrophy and deformation. Disk repositioning should be considered only when the disk is minimally deformed and of near-normal length (10).

Closed lock refers to an acute or chronic limitation of movement of the condyle owing to an intraarticular disturbance. Patients experiencing closed lock often complain of muscle dysfunction secondary to efforts to reach a baseline mouth opening. Arthrocentesis followed by arthroscopic lysis of superior joint space adhesions, la-vage, and manipulation are the treatments of choice for this condition. Open surgical procedures are indicated when arthroscopy has failed to resolve the restriction in opening. The choice of open procedure largely depends on disk anatomy and position (10).

In 1957, Henny and Baldridge introduced the technique of high condylectomy. Several authors reported in the literature the therapeutic success rate of the disc repositioning technique in conjunction with bone recontouring, with a range from 77% to 100%. Excellent results, with a success rate of 90%, were reported in 1985 by Dolwick and Sanders in a group of patients who underwent disc repositioning and condylar reduction. Walker and Kalamchi in 1987 show the good results with a surgical technique including the removal of 2-4 mm of the top of the condyle together with suturing of the disk on the toop of the condylar stumpand to the lateral capsule (11).

High condylectomy decreases the vertical dimension of the condyle; for this reason, some surgeons perform it bilaterally when the joint is not symptomatic and there is not any pathology in the joint (11). In our study population, the unilateral decrease of the condyle vertical dimension would lead to unbalanced occlusion and deviation. On the basis of this idea, eventhough the operations were performed bilaterally and in hence vertical dimension of the both condyles was decreased with surgical procedures, the deviation interestingly remained unchanged and it was present in all groups as before surgery.

In the analysis of response to surgical procedure of 3 different groups of patients, all findings beside devation had significant improvement postoperatively. But it was statistically insignificant difference between groups.

Bilateral surgical approach for TMJ is certain procedures with the respect of prognosis. The condition of patients with bilateral TMD with different diagnosis for each site or same diagnosis for both site don not present negative effect with the treatment and satisfaction for the prognosis. This study had a significance with the respect of prognosis of the bilateral TMD.

The improvement for maximal mouth opening postoperatively in the 1st, 2nd and 3rd group was 3.5mm, 4.1mm and 6.07mm, respectively. The scores for improvement in mouth opening was low, due to the satisfactory improvement for the other criteria, low improvement in mouth opening had become insignificant for the succesfull outcomes of the patient satisfaction. Marked improvement was provided in presence of pain, clicking, crepitation, headache. Patient satisfaction was high in all the groups. The operative procedures had positive affects on these clinical findings.

These succesfull results on patient satisfaction and TMJ surgical procedures was obtained though the presence of different diagnosis on the patients’ groups. Therefore, basically the patient satisfaction mainly depend on the basic surgical procedures. This study also encourage the patient and surgeon to manage the bilateral TMD.

This study verified that high condylectomy with or without additional procedures for the treatment of ADDR and ADDNR can improve the presence of pain, deviation, clicking, headache, maximal mouth opening, crepitation, however the deviation at rest was not significantly changed.
